# Durability monitoring of long-lasting insecticidal (mosquito) nets (LLINs) in Madagascar: physical integrity and insecticidal activity

**DOI:** 10.1186/s13071-017-2419-7

**Published:** 2017-11-13

**Authors:** Sanjiarizaha Randriamaherijaona, Jacky Raharinjatovo, Sébastien Boyer

**Affiliations:** 1 Unité d’Entomologie Médicale, Institut Pasteur de Madagascar, 1274 Avaradoha, 101 Antananarivo, BP Madagascar; 2 Ecole doctorale Sciences de la vie et de l’environnement, Université d’Antananarivo, 101 Antananarivo, Madagascar; 3 Population Services International Madagascar, 7748 Ampefiloha, 101 Antananarivo, BP Madagascar; 4 Medical Entomology Platform, Institute Pasteur of Cambodia, 5 Preah Monivong Blvd (93), Phnom Penh, Cambodia

**Keywords:** Malaria, Long-lasting insecticide-treated nets, Fabric integrity, Bio-efficacy, Madagascar

## Abstract

**Background:**

Long-lasting insecticidal mosquito nets (LLINs) are highly effective for malaria prevention. However, it is also clear that durability monitoring is essential to predict when, post-distribution, a net population, no longer meets minimum WHO standards and needs to be replaced. Following a national distribution campaign in 2013, we tracked two durability indicators, physical integrity and bio-efficacy at six and 12 months post-distribution. While the loss of net integrity during this period was in line with expectations for a one-year net life, bio-efficacy results suggested that nets were losing insecticidal effect faster than expected. The rate of bio-efficacy loss varied significantly between different net brands.

**Methods:**

We tested 600 randomly selected LLINs, 200 from each of three net brands. Each brand came from different eco-epidemiological zones reflecting the original distribution scheme. Fabric integrity (size and number of holes) was quantified using the proportional hole index (pHI). A subsample of the nets, 134 new nets, 150 at six months and 124 at 12 months, were then tested for bio-efficacy using the World Health Organization (WHO) recommended method.

**Results:**

Three net types, Netprotect®, Royalsentry® and Yorkool®, were followed. After six months, 54%, 39% and 45%, respectively, showed visible loss of integrity. The median pHI by type was estimated to be one, zero and one respectively. The percentage of damaged nets increased after 12 months such that 83.5%, 74% and 68.5%, had holes. The median pHI for each brand of nets was 47.5, 47 and 23. No significant difference in the estimated pHI at either six or 12 months was observed. There was a statistically significant difference in the proportion of hole size category between the three brands (*χ* ^2^ = 15.761, *df* = 4, *P* = 0.003). In cone bio-assays, mortality of new Yorkool® nets was surprisingly low (48.6%), mortality was 90.2% and 91.3% for Netprotect® and Royalsentry® (*F*_(2, 131) _= 81.59, *P* < 0.0001), respectively. At 12 month use, all tested nets were below the WHO threshold for replacement.

**Conclusion:**

These findings suggest that there is a need for better net quality control before distribution. More frequent replacement of LLINs is probably not an option programmatically. Regardless of prior approval, LLIN durability monitoring for quality assessment as well as net loss following distribution is necessary to improve malaria control efforts.

**Electronic supplementary material:**

The online version of this article (10.1186/s13071-017-2419-7) contains supplementary material, which is available to authorized users.

## Background

The use of long-lasting insecticide treated nets (LLINs) is a key malaria prevention measure. Unlike conventional bed nets, LLINs kill susceptible *Anopheles* spp. vectors. LLINs use in sub-Saharan Africa has increased by as much as 30% since 2010 [1]. The World Health Organization (WHO) estimates that in this part of the world, 53% of the population at risk of malaria slept under an LLINs in 2015 (95% confidence interval [CI]: 50–57).

LLINs prevent human-mosquito contact [2] by creating both a physical and an insecticidal barrier which, theoretically, will remain in effect despite repeated washing (at least 20 times), and during extended use (up to three years of use under field conditions). However, there is mounting evidence that these assumptions about the effective life of LLINs in the field are overly optimistic in some settings. Therefore, WHO has invited the National Malaria Control Programmes (NMCP) such as the Ministry of Public Health of Madagascar to assess the durability of LLINs in operational conditions. The recommended design includes three indicators: bio-efficacy, a measure of insecticidal effect, fabric integrity, a measure of physical damage sustained by the net and coverage, the loss of nets from houses where they had previously been hung. Threshold values for these indicators, below which LLINs are judged to need replacement, provide a reference that can be used to assess net loss from a population of nets, viz. nets of the same type, distributed at the same time.

In Madagascar among the 23 million inhabitants, about 2 million confirmed malaria cases and 6000 deaths were reported in 2015 [1], which occur across different eco-epidemiological zones (Additional file 1). The two coastal regions exhibit hyperendemic patterns with transmission lasting all year in the East and more than six months per year in the West. In the South, the period of transmission is short and episodic. Fringe, Central Highlands and South are prone to outbreaks. In the fringe areas, i.e. at intermediate altitudes, the transmission pattern is seasonal, lasting from November to May (rainy season). In the Central Highlands, the transmission is unstable, and episodic or epidemic [3]. There are four principal malaria vectors: *Anopheles funestus*, *An. gambiae* (*s.s.*), *An. arabiensis* and *An. mascarensis* [4–6]. Recently, *An. coustani*, infected with *Plasmodium* spp., was identified in the Central Highlands. The infection could be considered *Plasmodium vivax* or *P. falciparum* [7].

The current strategy of the Malagasy NMCP is based on effective case management and vector control, using LLINs and indoor residual spraying (IRS). In 2015, more than 11 million nets were distributed in Madagascar [1]. Thus, LLIN coverage is thought to be relatively high, approaching the universal coverage target of one net for every two people of the population at risk as recommended by the WHO. Nets fulfilling the criteria of ≥ 80% mortality in cone test were still effective as described in the World Health Organization Pesticide Evaluation Scheme guidelines (WHOPES) [8]. However, concerns about premature loss of ITN bio-efficacy exist [9] have raised the question of whether the bio-efficacy of many of these nets is adequate (given that nets with reduced fabric integrity i.e. more holes) rely on insecticidal effect as a compensatory mechanism, a problem seen elsewhere. In Uganda, ITN bio-efficacy testing indicated that Permanet® 2.0 LLINs produced 74% functional mortality after two years of household use in rural conditions [10]. Similarly, in Ethiopia, Permanet® 2.0 LLINs, used for two years, showed 67–72% mortality against *Anopheles arabiensis* [11]. In Cambodia, only 73% of tested LLINs fulfil the WHO criteria against *An. dirus* (*s.s.*) susceptible strain [12].

To guide planning around the timing of LLIN campaigns to achieve sustainable impact, net programs support LLIN durability monitoring. In this study, we report on physical integrity and the bio-efficacy of three brands of nets distributed in 2013 in six districts of Madagascar.

## Methods

### Study areas

**Fig. 1:**
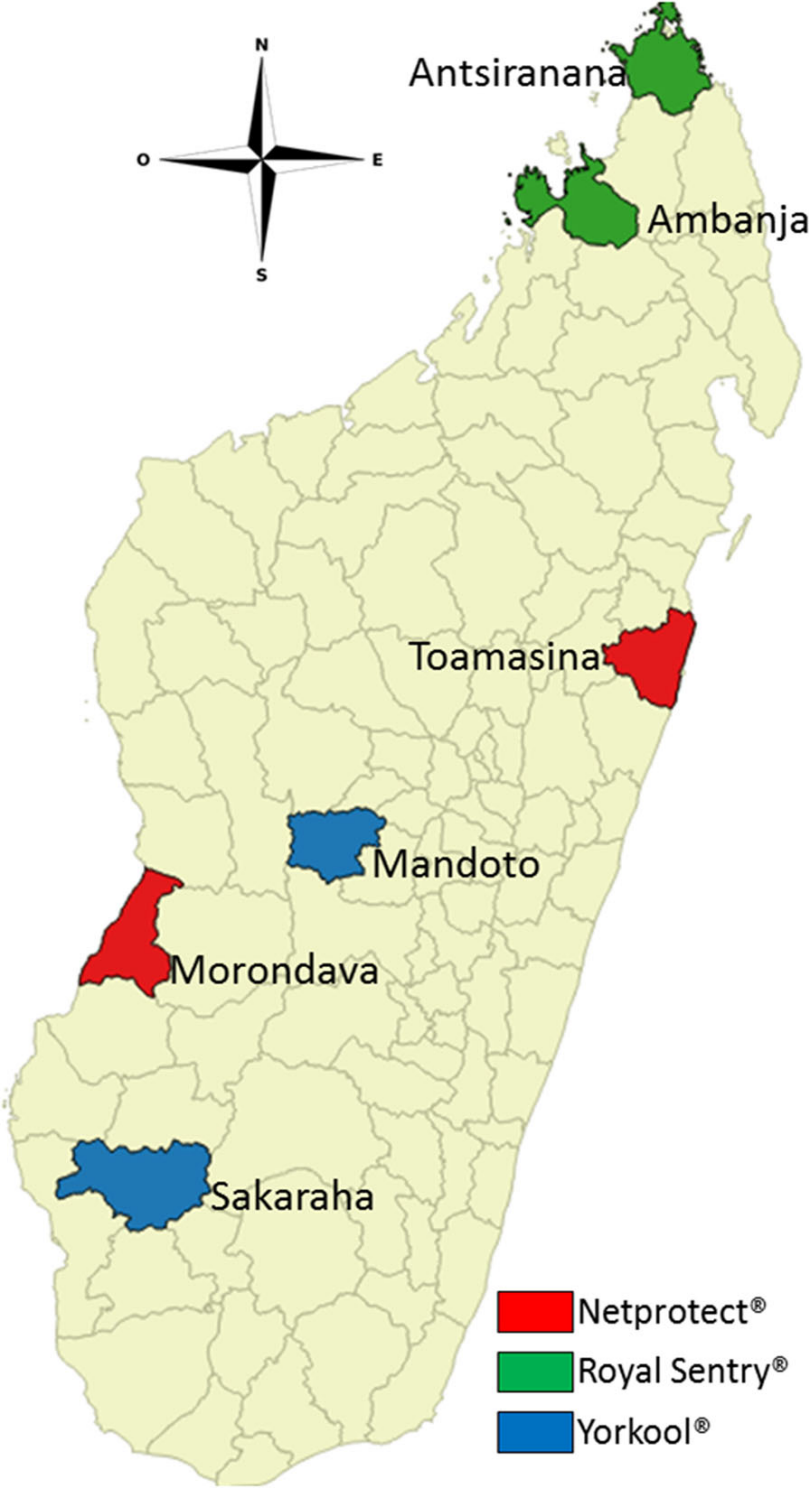
Study sites with different brand of nets

The study took place in six districts (Fig. 1). The study sites represent the environments and cultural settings in which LLINs were distributed during the mass campaign. Selected sites (Table 1) were chosen based on criteria such as epidemiology, LLIN brand distributed to local households and accessibility.

**Table 1:** Characteristics of net brands used during the study

Brands	Manufacturer	Thread	Insecticide	Concentration	Fabrication
Netprotect®	Best Net Europe LTD	Polyethylene	Deltamethrin	68 mg a.i./m^2^	Incorporated
Royalsentry®	Disease control Technologies LLC	Polyethylene	Alpha-cypermethrin	261 mg a.i./m^2^	Incorporated
Yorkool®	Yorkool International Company LTD	Polyester	Deltamethrin	55 mg a.i./m^2^	Coated

*Abbreviation*: *a.i.* active ingredient

### LLINs

Three LLINs brands followed during the study are described in Table 2. Netprotect® ITNs manufactured by Bestnet Europe LTD, is an LLIN made of polyethylene with deltamethrin incorporated into the 118-denier monofilament with a target dose of 1.8 g active ingredient (a.i.)/kg, corresponding to 68 mg a.i./m^2^. Royalsentry® ITNs manufactured by Disease control Technologies LLC, is an LLIN made of polyethylene with alpha-cypermethrin incorporated into the 150-denier monofilament with a target dose of 5.8 g (a.i.)/kg corresponding to 261 mg/m^2^. Yorkool® ITNs manufactured by Yorkool International Company LTD, is an ITN made of polyester fibre coated with deltamethrin 55 mg (a.i.)/m^2^. All LLINs tested were rectangular.

**Table 2:** Study sites with epidemiological characteristics

Characteristics	District	Netprotect®	Royalsentry®	Yorkool®
Perennial transmission	Toamasina	100 nets	–	–
	Ambanja	–	100 nets	
Long transmission sites	Morondava	100 nets	–	–
	Antsiranana	–	100 nets	–
Seasonal transmission	Mandoto	–	–	100 nets
	Sakaraha	–	–	100 nets

### Study design and sample size

A phase III field trial, designed as a prospective longitudinal study, was set up to study the performance of three brands of nets. There were two rounds of data collection during which the same LLINs were evaluated after six months and 12 months in the field (initial distribution occurred in late 2013). The 6-month survey was done from April to June 2014 for five sites (Ambanja, Morondava, Antsiranana, Mandoto and Sakaraha) and in July for Toamasina. Toamasina was different from the other sites because continuous LLIN distribution started there in January 2014. The 12 month (in 2014) surveys were conducted from September to November for the five sites and in February for Toamasina. This study adopted WHO guidelines for the minimum sample size required per product advised for better precision. During this study, 100 LLINs per site, selected randomly according to site accessibility, were used for physical integrity assessment using the WHO recommended hole assessment method [8]. A sub-sample of 30 LLINs per site/time point was randomly selected for further bio-efficacy test. Nets collected for the bioassays analysis were replaced with new nets at each point in time. Collected nets were labelled and stored in separate plastic bags which were transported to a central laboratory for physical integrity and bioassay testing.

A total of 128 LLINs newly removed from plastic storage bag, including Netprotect® (*n* = 40), Royalsentry® (*n* = 46) and Yorkool® (*n* = 48) were used to assess and develop a baseline, for LLIN bio-efficacy profile.

### LLIN physical integrity

LLINs sampled during the bio-efficacy assessment were scored for fabric-integrity (physical integrity). Integrity was quantified by as described in WHOPES [8]. Hole sizes were categorised into four groups; holes smaller than a thumb (0.5–2.0 cm), holes between a thumb and a closed fist (2–10 cm), holes between a closed fist and a head (10–25 cm) and holes bigger than a head (> 25 cm). A proportionate hole index (pHI), which characterises the midpoint diameter to an estimated hole size [13–15], was calculated by making the sum of the holes weighted by size for each net. For these groups, the weights used to calculate the pHI were 1, 23, 196 and 576. To better translate the hole index to an integrity status (net condition) for each sampled net, the pHI is categorised into “good” (0–64), “damaged” (65–642), and “so torn” that protection from mosquitoes was judged to be compromised (≥ 643) [8].

### ITN bio-efficacy assessment: WHO cone test method

**Fig. 2:**
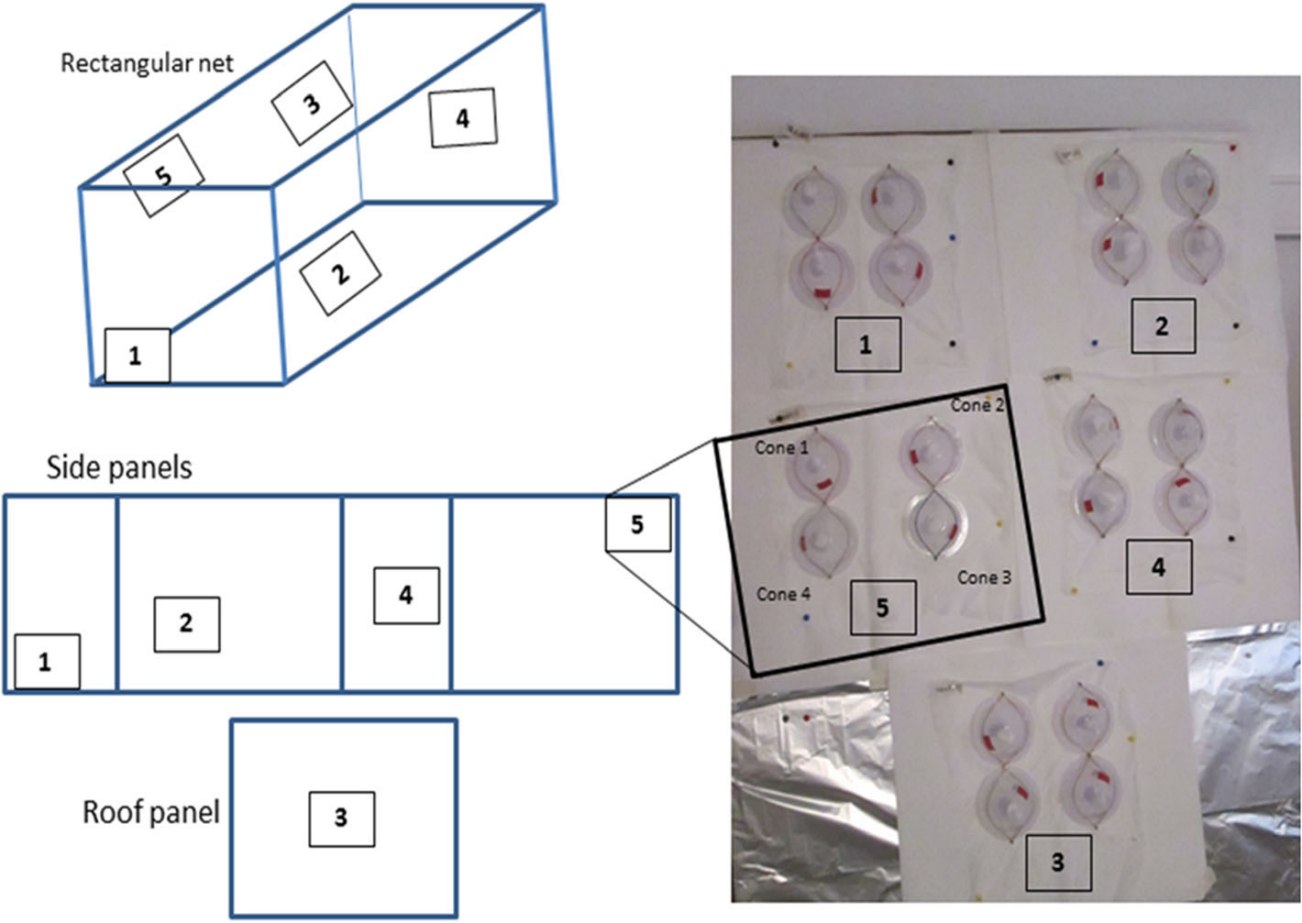
Sampling locations used for a rectangular type bed net and bioassay

Standard WHO cone bioassays were performed with a susceptible laboratory strain of *Anopheles arabiensis* [16], following the recommendations of the WHOPES [8]. For each LLIN, five 30″ × 30″ sub-samples were cut from the LLIN selected for testing. The subsamples were cut from the top and each of the four sides of the net. Each sub-sample was placed in an aluminium foil envelope, labelled, and kept individually in a 4 °C refrigerator before conducting the bio-assay. For each sub-sample, four cone tests were conducted at a time following standard WHO procedure [8] (Fig. 2). Five non-blood-fed, two-to-five-day-old female *An. arabiensis* were introduced into each cone and exposed to LLIN samples for 3 min, before being transferred to paper cups, covered with netting, and held for 24 h at 28 °C and 80% humidity with access to 10% sugar solution. Mortality was recorded 24 h post-exposure. By following this methodology, a total of 100 mosquitoes were tested for each net. Each day of testing, four cones, each with 10 *An. arabiensis* were fixed on a non-impregnated net as a negative control. If the mortality in the control was < 10% for a given day, the data were adjusted with Abbott’s formula [17]. If the mortality in the control was > 10%, all the tests for that day were repeated. The standard protocol recommends using a mixed outcome, i.e. mortality ≥ 80% or KD ≥ 95% to consider a net as effective. Results from a study on nets bioassay suggest that mortality outcome was better than the KD outcome at predicting the validity of LLINs.

### Data analysis

All data were recorded on standard forms, before being entered into an Excel database and then imported into R, version R-3.1.3, for statistical analysis [18]. Categorical variables were compared using Chi-square test, and continuous, discrete variables were tested using Analysis of Variance (ANOVA) to test the significance among the three study areas or the three brands of nets. A *P*-value < 0.05 was considered significant. The Tukey's HSD test was applied to assess the significance of the differences.

## Results

### Physical condition of LLINs

**Fig. 3:**
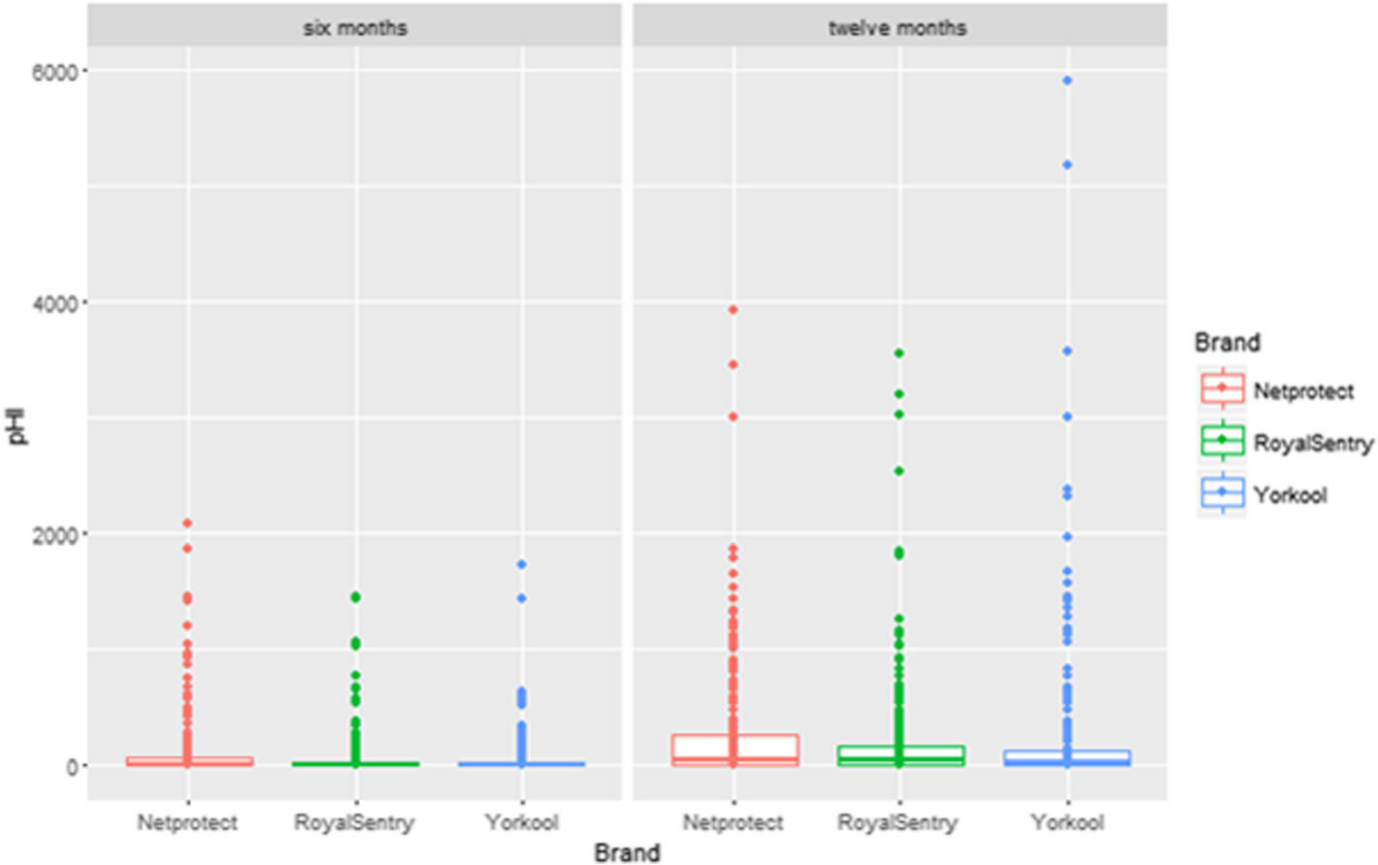
Median and interquartile range (IQR 0.25–0.75) of proportionate hole index (pHI)

A total of 600 nets, 200 for each of the three brands, were sampled and scored for integrity six months after distribution and hanging. There were 47%, 62% and 46% of nets examined were completely intact (no holes) for Netprotect®, Royalsentry® and Yorkool®, respectively. The percentages of intact nets dropped to 16.5% (*n* = 200), 26% (*n* = 200) and 31.5% (*n* = 200) for Netprotect®, Royalsentry® and Yorkool®, respectively, at 12 months post-distribution (Table 3). The median and interquartile range (IQR 0.25–0.75) was used to present the pHI in Fig. 3 and Table 4. After six months of use, the median pHI was 1 (IQR 0–66.25) for Netprotect®, 0 (IQR 0–27) for Royalsentry® and 1 (IQR 0–25) for Yorkool®. In Toamasina and Morondava, where Netprotect® was distributed and in Antsiranana and Ambanja where Royalsentry® was distributed, significant difference of pHI median values was observed (*F*_(1,198) _= 5.08, *P* = 0.02) (Table 5). After 12 months, the pHI increased to 47.5 (IQR 2–271.2), 47 (IQR 0–162.5) and 23 (IQR 0–123) for Netprotect®, Royalsentry® and Yorkool®, respectively. The median and interquartile range for each district are shown in Table 4.

**Table 3:** Percentage of unholed nets

Follow-up time	District	6 months		12 months	
		*n*	%	*n*	%
Netprotect®	Morondava	100	33	100	19
	Toamasina	100	60	100	14
Royalsentry®	Ambanja	100	80	100	30
	Antsiranana	100	43	100	22
Yorkool®	Mandoto	100	47	100	28
	Sakaraha	100	44	100	35

**Table 4:** Median and interquartile range (IQR 0.25–0.75) of proportionate hole index (pHI)

Net brand	Months	*n*	Average pHI	Median pHI	Interquartile range
Netprotect®	6	200	121.5	1	0–66.25
	12	200	281.5	47.5	2–271.2
Royalsentry®	6	200	79.65	0	0–27
	12	200	220.9	47	0–162.5
Yorkool®	6	200	71.64	1	0–25
	12	200	271	23	0–121

**Table 5:** Median and interquartile range (IQR 0.25–0.75) of proportionate hole index (pHI) by locality

Net brand	District	Months	*n*	Average pHI	Median pHI	Interquartile range
Netprotect®	Toamasina	6	100	81.47	0^a^	0–34
		12	100	279.6	46	2–271.8
	Morondava	6	100	163.1	23	0–77
		12	100	283.2	50	2–256.2
Royalsentry®	Antsiranana	6	100	114.2	23^b^	0–77
		12	100	242.5	51.5	4–193.5
	Ambanja	6	100	44.75	0	0
		12	100	199	28	0–127
Yorkool®	Mandoto	6	100	54.3	1	0–25
		12	100	355.2	23	0–164.2
	Sakaraha	6	100	88.99	1	0–25
		12	100	187.7	23	0–97

^a^Significant difference between districts with the same brand nets (*F*_(1, 198)_ = 3.45, *P* = 0.05)

^b^Significant difference between districts with the same brand nets (*F*_(1, 198)_ = 5.08, *P* = 0.02)

At six months, the mean pHI for polyethylene and polyester nets was 71.6 and 101, respectively. At 12 months, this increased to 271 and 251 for polyethylene and polyester nets, respectively. There was no significant statistical difference between net threads neither at six or 12 months.

The proportion of LLINs judged to be in “good”, “damaged”, or “too torn” categories at different ages of follow-up are summarised in Table 6. After six months use, more than 70% of distributed nets from the three bands were in “good” condition. At 12 months post-distribution, 55.6%, 56.8% and 69.2% of Netprotect®, Royalsentry® and Yorkool®, respectively, were in “good” condition. There was a statistically significant difference in the proportion of hole size category between the three brands (*χ* ^2^ = 15.761, *df* = 4, *P* = 0.003) with Yorkool® showing greater loss of integrity followed by NetProtect® and RoyalSentry®.

**Table 6:** Physical condition of nets by locality

LLIN physical condition			Good		Damaged		Too torn	
	District	*n*	6 months	12 months	6 months	12 months	6 months	12 months
Netprotect®	Toamasina	100	78	58	20	28	2	14
	Morondava	100	70	53	21	31	9	16
Royalsentry®	Antsiranana	100	72	52	22	39	6	9
	Ambanja	100	92	61	5	29	3	9
Yorkool®	Mandoto	100	87	68	12	18	1	14
	Sakaraha	100	79	70	20	20	1	10

### Bio-efficacy

Results from WHO cone bioassays are presented in Table 7. Mortality in the negative control never exceeded 2%.

**Table 7:** Bio-efficacy results comparing three LLINs products. There was no significant difference between values which share the same letters

	Netprotect®		Royalsentry®		Yorkool®	
	*n*	Mortality	*n*	Mortality	*n*	Mortality
Beseline	40	91.1^a^	46	90.2^a^	48	48.6^b^
6 months	60	37.4^c^	60	32.0^d^	30	23.1^e^
12 months	38	11.0^f^	47	23.1^g^	39	14.0^f^

**Fig. 4:**
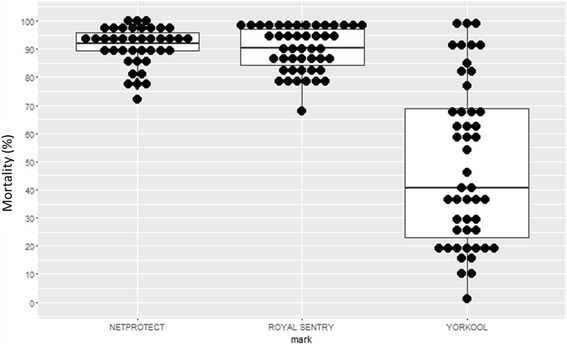
Bio-efficacy results on baseline nets

At baseline, Yorkool® LLINs were already “not fully effective” according to the threshold established by WHO (mortality < 80%). There was a significant difference in mortality between the three net brands (*F*_(2, 131) _= 81.59, *P* < 0.0001). Significant difference between Yorkool® and Netprotect®, both of which were treated with deltamethrin, was observed (Tukey's HSD test, *P* < 0.0001). However, there was no statistical difference between mortality induced by Netprotect® and Royal Sentry®. New Netprotect® LLINs caused an average mortality of 91% with 90% (36/40) being above the minimum threshold. For Royalsentry®, the average induced mortality was 90%, and 91.3% (42/46) were above the threshold for acceptable insecticidal effect. Yorkool®, in contrast, presented the lowest induced mortality. The average mortality was 48.6%, with only 20.8% (10/38) being above the minimum threshold (Fig. 4). After six months, mortality decreased significantly for all three net types enrolled in the assessment (*F*_(2, 147) _= 6.33, *P* = 0.002). The average mortality was 37.4% for Netprotect®, 32 and 23.1%, respectively, for Royalsentry® and Yorkool ®. Only one Royalsentry® and one Netprotect® net scored above the threshold level; none of the Yorkool® nets tested was found to be above the minimum threshold. The difference between Royalsentry® and Netprotect®, both made of polyethylene, was not significant (Tukey's HSD test, *P* = 0.23). Even though both are treated with the same pyrethroid insecticide, deltamethrin, Yorkool® and Netprotect® LLINs caused significantly different mortality rates (Tukey's HSD test, *P* = 0.001). No significant difference was noted between mortality caused by Yorkool® and Royalsentry® (Tukey HSD, *P* = 0.07).

At 12 months, the mortality associated with all LLINs tested decreased considerably. For LLINs treated with deltamethrin (Netprotect® and Yorkool®), the average mortality rates were 11% and 14%, respectively, with no significant difference between those brand nets. Alpha-cypermethrin-treated LLINs (Royalsentry®) showed an average mortality rate of 23.1%. A significant difference was observed between Royalsentry® - Netprotect® and Royalsentry® - Yorkool®. However, none of the 12-month nets exceeded the minimum threshold characteristic of nets described as “in need of replacement”.

**Fig. 5:**
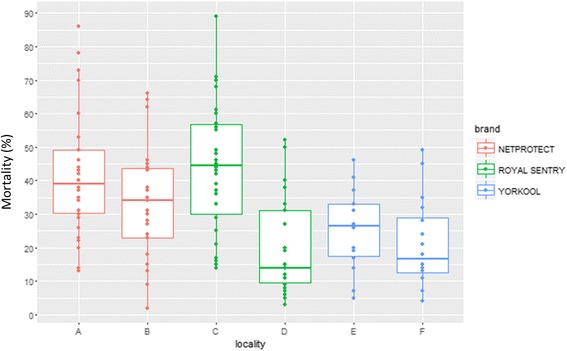
Bio-efficacy results on six months of use, by locality. Localities: A, Toamasina (*n* = 30); B, Morondava (*n* = 30); C, Antsiranana (*n* = 30); D, Ambanja (*n* = 30); E, Mandoto (*n* = 21); F, Sakaraha (*n* = 21)

**Fig. 6:**
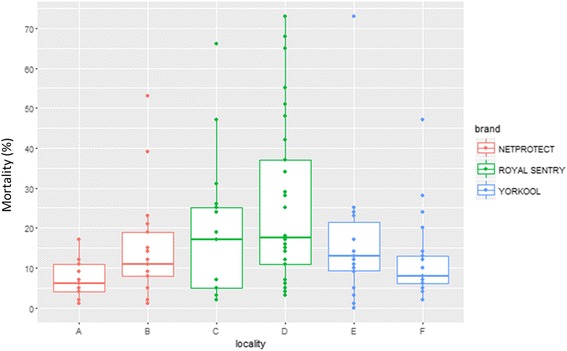
Bio-efficacy results on 12 months of use, by locality. Localities: A, Toamasina (*n* = 17); B, Morondava (*n* = 21); C, Antsiranana (*n* = 17); D, Ambanja (*n* = 30); E, Mandoto (*n* = 18); F, Sakaraha (*n* = 21)

Differences in mortality associated with the location of the LLIN were also observed. At six months, there was a significant difference between percent mortality of Netprotect® distributed in Toamasina and Morondava (*F*_(1, 58)_ = 4.18, *P* = 0.04), 41.9 *vs* 33%, respectively. In Antsiranana and Ambanja, where Royalsentry® was distributed, mortalities were 43.8 and 19.7%, respectively (*F*_(1, 58)_= 32.49, *P* < 0.0001). In Mandoto and Sakaraha, where Yorkool® was distributed, mortality was 25.4 and 21.1%, respectively (*F*_(1, 28 )_ = 0.83, *P* = 0.37) (Table 8, Fig. 5). At 12 months, the average mortality rate for all three brands ranged between 6.9 and 25.9% (Fig. 6). No significant difference was found between the values (Table 8).

**Table 8:** Bio-efficacy of LLINs products by locality

Follow-up time		6 months		12 months	
		No. of tested nets	Average mortality (%)	No. of tested nets	Average mortality (%)
Netprotect®	Toamasina	30	41.9^a^	17	6.9
	Morondava	30	33	21	14.3
Royalsentry®	Antsiranana	30	43.8^b^	17	18.1
	Ambanja	30	19.7	30	25.9
Yorkool®	Mandoto	14	25.4	18	16.2
	Sakaraha	16	21.1	21	12.1

^a^Significant difference between districts with the same brand nets (*F*_(1, 58)_ = 4.18, *P* = 0.04)

^b^Significant difference between districts with the same brand nets (*F*_(1, 58)_ = 32.49, *P* < 0.0001)

## Discussion

This study is one of only a few studies that examine bio-efficacy as well as fabric integrity under operational conditions in Madagascar.

In the present assessment, more than half of nets were still in a good physical condition after 12 months use. This result parallels observations made in an earlier (2012) investigation involving epidemiological and entomological methods in the South-East region of Madagascar, to identify factors that could have caused a malaria outbreak. Among 39 LLINs collected during the study, 15%, 42.5% and 42.5% were observed to be “good”, “damaged” and “too torn” condition, respectively, after two years of use [19].

For LLINs made of polyester, the percentage of nets having any holes after 12 months was higher than in Western Uganda where 33.7% of nets were assessed as having holes after one year [20]. In Zambia, 9.6% of polyester and polyethylene nets were classified as “too torn” after 12 months in the field [21], which is a relatively low proportion compared to observation in this study where 11.9% of polyester nets and 12.1% of polyethylene nets were torn. There is evidence that at the household level, LLINs can inhibit blood feeding even when they are in damaged condition (65 < pHI < 642). This is partially due to the repellent effect of pyrethroids incorporated or coated on the net [20]. Nonetheless, a high value of pHI (> 643), would easily allow for a mosquito to enter an LLIN to bite a sleeping human and then take rest outside the LLIN.

The findings on net bioassays were surprising, especially given that most programs assume that LLINs retain their insecticidal activity for at least three years [22]. At the baseline of the current study, 10% of new Netprotect® nets and more than 75% of new Yorkool® nets did not meet the WHO cut-off value. The proportion of new nets which did not meet the WHO criteria was probably due to a problem in the manufacturing process. In Cambodia, 100% of Netprotect® used as baseline met the WHOPES criteria but, 43% of them had a deltamethrin content below the target dose [23]. These results suggest that cone bioassays alone might be not adequate to assess the comparative efficacy of these nets. Therefore, more elaborate tests such as a ring-net bioassay, which measures the median knockdown time (MKDT) of mosquitoes on three different LLINs should be performed to understand the bioavailability of the insecticide on the LLINs. The MKDT is expected to be directly correlated to the insecticide concentration on the surface for fast acting pyrethroids. It could be helpful in assessing the amount of insecticide left on the net thread [34]. Added to that, tunnel test was not done here due to lack of materials. Such a test is recommended by the WHOPES, for each net that fails to meet the criteria of the WHO cone test [3].

Bioassay results from this current study showed one very low mortality rate at six and 12 months uses. At 12 months, average percent mortality was 11%, 14% and 23% for Netprotect®, Yorkool® and Royalsentry®, respectively. However, other studies in another country showed that at 12 months, the average percent of mortality could be relatively high depending on net type [21, 24, 25]. A significant difference of percent mortality between two localities after six months may be explained by user behavior. In Madagascar, some reasons could lead the household to frequently use or not-use of bed net, for example the feeling of suffocation during the night, the skin irritation due to insecticide on the net, the room becomes darker [26].

The highest proportion of nets that needed replacement was found in an area where Netprotect® was distributed; 52.6% (10/19) of them were too torn and whose protective bio-efficacy for the user was in serious doubt. In this study, the finding was that more than 90% and 84% of nets collected after six and 12 months were in good or damaged condition but did not meet the WHO bioassay cut-off value. These results are important to better understand the link between the standard measure of holes, by weighting the number of each hole size and insecticide bio-availability.

The results from this study could have several implications for the LLIN strategy of the NMCP. The finding was that percent mortality induced by Yorkool® was lower than those induced by Netprotect® and Royalsentry® even if newly removed from plastic storage bag. This information may be relevant to the Malagasy NMCP regarding the effectiveness of LLINs brand, based on bio-efficacy, for future LLIN mass distribution through campaigns. Our findings are limited to the three LLIN brands, Netprotect®, Royalsentry® and Yorkool®, distributed during the mass campaign in 2013; other LLIN brands could perform differently under the same or different conditions. Hence, results from this study may not be extrapolated to other LLIN brands, even in similar settings. Moreover, more research still needs to be conducted to determine how the physical integrity and the residual bio-efficacy, of any brand, affect its skill to prevent and reduce malaria transmission. In the one hand, monitoring of new nets is needed before an LLIN mass campaign, on their arrivals in port. Before starting such mass distribution, it would be essential that nets are checked for their compliance with WHO specifications. Results from this study also show the importance of the quality control along the supply chain, right through hanging of the LLIN. On the other hand, the manufacturers need to clarify the quality-assessment/quality-control (QA/QC) of their product and the guarantee to prospective LLIN customers of the “performance” of their products for three years. This guarantee is currently based on laboratory results from insecticidal activity and burst strength tests [8]. As more field monitoring of LLINs durability is conducted by public or private institutions, more evidence of LLIN insecticidal activity will inform these changes needed.

## Conclusion

This study is the first to report on the performance of nets under operational conditions in Madagascar by checking the physical integrity and the insecticidal efficacy of new nets and nets post distribution. The findings from this study highlight the low insecticide efficacy of Yorkool® even if unused. This recommends that there is a need for better net quality control before LLIN mass deployment.

## Additional file

Additional file 1: Malaria transmission patterns in the districts of Madagascar (TIFF 310 kb)

## Abbreviations

ANOVA: analysis of variance
CI: confidence interval
IQR: interquartile range
ITN: insecticide treated net
KD: knock-down
LLINs: long-lasting insecticidal nets
MKDT: median knock-down time
NMCP: National Malaria Control Programme
pHI: proportional hole index
QA/QC: quality-assessment/quality-control
WHO: World Health Organization
WHOPES: World Health Organization pesticide evaluation scheme
