# Supplementary figures and images for: Durability monitoring of long-lasting insecticidal (mosquito) nets (LLINs) in Madagascar: physical integrity and insecticidal activity
Source: Parasit Vectors. 2017 Nov 13;10:564. doi: 10.1186/s13071-017-2419-7 (PMC5683549; doi:10.1186/s13071-017-2419-7)

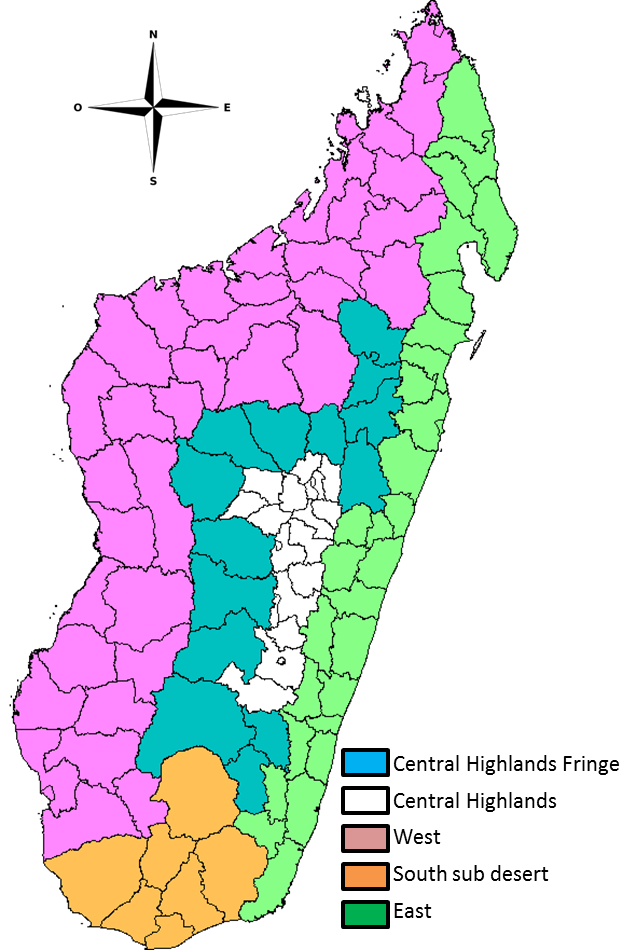

Supplement: Additional file 1: — Malaria transmission patterns in the districts of Madagascar (TIFF 310 kb) [file 13071_2017_2419_MOESM1_ESM.tif]
